# Electrically tunable strong coupling in a hybrid-2D excitonic metasurface for optical modulation

**DOI:** 10.1038/s41377-025-02079-3

**Published:** 2026-01-02

**Authors:** Tom Hoekstra, Jorik van de Groep

**Affiliations:** Van der Waals-Zeeman Institute, Institute of Physics, University of Amsterdam, Amsterdam, the Netherlands

**Keywords:** Metamaterials, Photonic devices

## Abstract

Atomically thin semiconductors exhibit tunable exciton resonances that can be harnessed for dynamic manipulation of visible light in ultra-compact metadevices. However, the rapid nonradiative decay and dephasing of excitons at room temperature limit current active excitonic metasurfaces to a few-percent efficiencies. Here, we leverage the combined merits of pristine 2D heterostructures and non-local dielectric metasurfaces to enhance the excitonic light-matter interaction, achieving strong and electrically tunable exciton-photon coupling at ambient conditions in a hybrid-2D excitonic metasurface. Using this, we realize a free-space optical modulator and experimentally demonstrate 9.9 dB of reflectance modulation. The electro-optic response, characterized by a continuous transition from strong to weak coupling, is mediated by gating-induced variations in the free carrier concentration, altering the exciton’s nonradiative decay rate. These results highlight how hybrid-2D excitonic metasurfaces offer novel opportunities to realize nanophotonic devices for active wavefront manipulation and optical communication.

## Abstract (web summary)

Electrically tunable strong coupling is achieved at ambient conditions in an excitonic metasurface, enabling 9.9 dB of free-space optical modulation.

## Graphical abstract

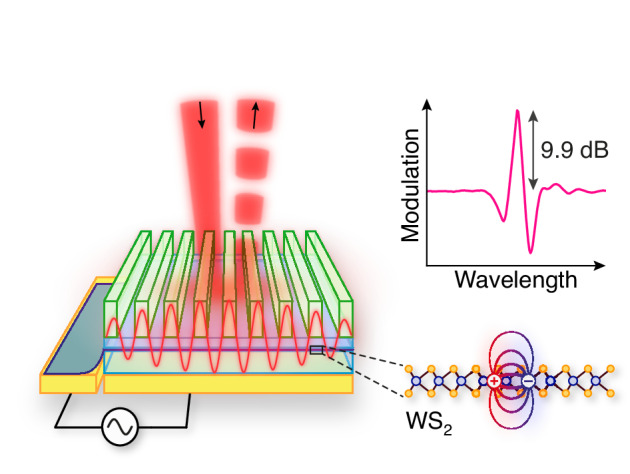

## Introduction

Van der Waals materials and their heterostructures have emerged as a transformative materials platform in photonics, as the layered 2D structure and lack of dangling bonds offer atomic-level control over thickness in arbitrary stacking configurations^1,2^. Combined with properties such as high refractive indices, large anisotropies, and broad tunability, the prospects of two-dimensional (2D) materials in nanophotonics research are tantalizing. In particular, monolayer 2D semiconductors stand out for their uniquely strong exciton resonances, which dominate their room-temperature optical properties due to the exceptionally large binding energies arising from quantum- and dielectric confinement in the atomically thin crystal^3^. Additionally, these excitons are highly sensitive to external stimuli^4–8^, with free-carrier injection emerging as an attractive tuning mechanism with potential optoelectronic applications including light detection and ranging^9^, free-space optical communications^10^, and analog image processing^11^.

Efficient electrical tunability, as observed in monolayer 2D semiconductors, would be an enormous asset in the field of metasurfaces. Already, these ultra-compact optical coatings provide remarkable new ways to manipulate light by harnessing the strong light-matter interactions offered by resonant metallic or dielectric nanostructures^12–16^. Yet, so far, dynamic metasurfaces have been few and far between due to the weak tunability of plasmonic and geometric resonances^10^. While a variety of active devices have already been demonstrated^17–21^, they are typically hindered by small modulation depths, excessive complexity, or operation limited to the infrared spectral range. As such, it seems natural to capitalize on the large tunability of excitons in 2D materials. Indeed, tunable excitonic metasurfaces have already been created by directly carving zone plate lenses and metagratings out of monolayers^22,23^. However, their efficiencies are restricted to a few percent because of the atomic thickness as well as severe damping of the exciton transition at ambient conditions by nonradiative decay channels^4^ and dephasing^24^. It is therefore essential to find ways of enhancing the excitonic light-matter interaction strength.

To this end, 2D materials have been placed in the vicinity of optically resonant nanostructures to leverage their strong Purcell enhancements^25–29^. In particular, a monolayer semiconductor was recently integrated in a plasmonic metasurface to achieve 10% reflectance modulation as well as dynamic beam steering^30^. Nevertheless, the observed performance was still constrained by defects and disorder in the unprotected monolayer arising from incompatibilities with standard nanofabrication protocols^31^. At the same time, it is well-established that 2D materials can be protected in heterostructures by encapsulating them with hexagonal boron nitride (hBN). This additionally provides screening from local charge fluctuations and defects in the dielectric environment, resulting in pristine excitonic properties^32,33^. Even with relatively simple planar heterostructures, this has led to remarkable realizations of near-perfect excitonic absorption^24^, near-unity reflection modulation^34,35^, and active beam steering devices^36,37^, although only at cryogenic temperatures. Hence, it is worthwhile to consider hybridizing heterostructures with metasurfaces^38,39^ as a novel route towards active light manipulation beyond the cryogenic regime.

Here, we combine the pristine excitonic properties of a Van der Waals heterostructure with the nanophotonic field enhancement offered by a non-local metasurface to demonstrate a dynamically tunable excitonic metasurface with high efficiencies. This hybrid-2D configuration enables strong exciton-photon interactions, which we harness for free-space optical modulation by electrically manipulating excitons in monolayer tungsten disulfide (WS_2_). By switching between the weak and strong coupling regimes, we demonstrate 9.9 dB modulation of the reflected beam at room temperature, which is a fivefold improvement over the previous record reflection contrast achieved with excitonic 2D materials. Furthermore, we show that the gating-induced free carrier density governs the exciton’s nonradiative decay rate, providing a continuous transition from strong to weak exciton-photon coupling. Altogether, these results demonstrate that, by leveraging the combined merits of dielectric metasurfaces and Van der Waals heterostructures, electrically tunable hybrid-2D metasurfaces hold the key to realize ultracompact optoelectronic devices for dynamic wavefront manipulation at ambient conditions.

## Results

### Hybrid-2D optical modulator

**Fig. 1:**
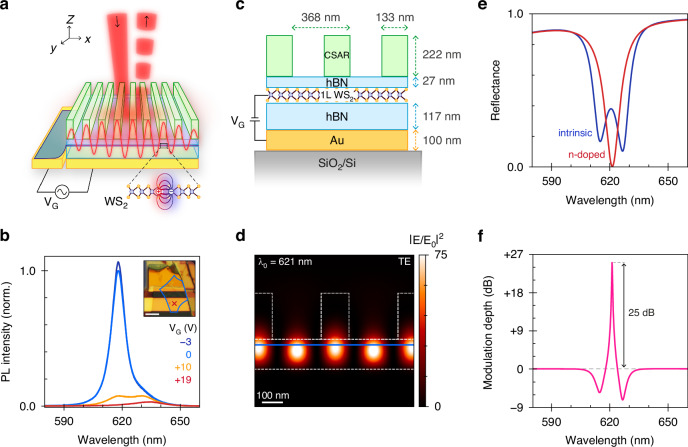
Hybrid-2D optical modulator. **a** Illustration of the concept: the electrical tunability of a monolayer 2D semiconductor in a heterostructure cavity is combined with the strong light-matter interaction of a dielectric non-local metasurface to achieve strong intensity modulation of the reflected beam. **b** Experimentally measured photoluminescence (PL) intensity for a bare heterostructure cavity as a function of gate voltage (color). The inset shows a micrograph of the heterostructure, with the monolayer outlined in blue and the measurement position indicated by the red cross. Scalebar: 5 μm. **c** Schematic of the detailed geometry of the modulator design (not to scale). **d** Electric field intensity enhancement for a transverse electric (TE) polarized normal-incident wave at free-space wavelength λ_0_ = 621 nm. The blue line represents the monolayer. **e** Calculated reflectance spectra for the device with an intrinsic (*R*_i_, blue) and n-doped (*R*_n_, red) monolayer. **f** The corresponding modulation depth (10⋅log_10_ (*R*_i_/*R*_n_), magenta) extracted from **e**

Figure 1a illustrates a free-space optical modulator whose performance is derived from the combined merits of monolayer 2D semiconductors and dielectric metasurfaces. The hybrid-2D modulator achieves strong modulation of the input field through the electrical tunability of exciton resonances in atomically thin tungsten disulfide (WS_2_) combined with the electromagnetic field enhancement offered by guided-mode resonances^30,40^. Applying a voltage between the gold (Au) back-gate and the monolayer results in strong electron-doping of the WS_2_, which increases the exciton-electron (Coulomb) scattering rate and thereby efficiently quenches the so-called A-exciton transition^5,6^. To demonstrate this, we measure the excitonic photoluminescence emission in a flat reference device at room temperature and show a 108-fold reduction at *λ*_*0*_ = 618 nm, while the charged exciton^41^ (trion) peak at 635 nm remains mostly unaltered (Fig. 1b). Although electrical tuning of exciton emission is by now well-established, such efficient quenching has thus far mostly been limited to cryogenic temperatures^24,34,35^.

The designed optical modulator combines several key elements to leverage the tunability of 2D excitons (Fig. 1c). First, the WS_2_ monolayer is electrically connected to the ground electrode, but otherwise isolated through encapsulation with hexagonal boron nitride (hBN). This provides screening from local charge fluctuations and defects in the dielectric environment, resulting in a narrow exciton linewidth^33^. At the same time, the resulting Van der Waals heterostructure, combined with the Au back-reflector, functions as an asymmetric optical cavity to confine light inside the layer stack^24^. Second, to maximize the light-matter interaction, we introduce a dielectric subwavelength grating on top of the heterostructure to enable resonant excitation of its fundamental TE_0_ guided mode. The resulting guided mode resonance (GMR) provides a 74-fold electric field intensity enhancement and greatly increases the interaction length of light with the WS_2_ monolayer (Fig. 1d). Finally, the back-reflector additionally serves as a local back-gate to the WS_2_ layer, where the bottom hBN layer functions as the gate dielectric. As such, the back-gate enables electrical manipulation of the carrier concentration in the monolayer. In the strongly electron-doped (n-doped) regime, the exciton is effectively quenched, and the reflectance spectrum shows a single critically coupled resonance, splitting into two strongly coupled resonances when the monolayer is brought to intrinsic doping instead (Fig. 1e). Consequently, in simulation, the device exhibits an absolute reflectance contrast of *ΔR* = 47.5% (*λ*_0_ = 627 nm) when it is switched between these two states. We quantify the modulation depth as 10log_10_(*R*_i_/*R*_n_), where *R*_i_ and *R*_n_ are the reflectance in the intrinsic and n-doped regimes, respectively (Fig. 1f). Using this definition, our modulator achieves 25 dB peak signal modulation at the operating wavelength.

### Tailoring the design for strong light-matter interactions

Next, we outline our approach for maximizing the modulation efficiency by simultaneously designing for perfect absorption in the exciton-quenched state and strong exciton-photon coupling in the unquenched state. To this end, we use rigorous coupled-wave analysis (RCWA) simulations to optimize the dimensions of the heterostructure cavity as well as the grating’s unit cell. Here, we choose to design the modulator for operation around the A-exciton wavelength, *λ*_A_ = 620 nm, with normal-incident TE-polarized light, while retaining a mirror-like response for TM polarization.

**Fig. 2:**
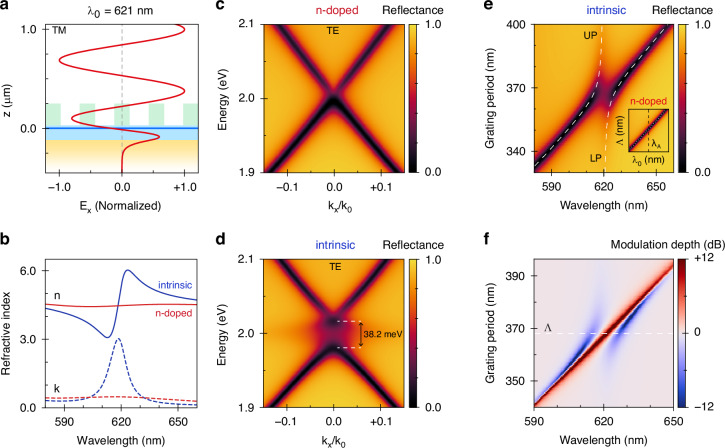
Nanophotonic design of the hybrid-2D metasurface. **a** Standing wave profile of the electric field under normal-incidence transverse magnetic (TM)-polarized illumination showing minimal field overlap with the monolayer (blue line at z = 0 μm). **b** Modeled real (n, solid) and imaginary (k, dashed) components of the complex refractive index of monolayer WS_2_ at intrinsic (blue) and strong n-type (red) doping. **c** Numerically simulated angular dispersion of the designed modulator under TE-polarized illumination with monolayer WS_2_ at strong n-type doping and **d** intrinsic doping. In the latter case, the A-exciton is strongly coupled to the GMR, resulting in the formation of a 38.2 meV gap (indicated) at the exciton energy. **e** Grating period dependence of the reflectance at intrinsic doping and strong n-type doping (inset). The calculated upper (UP) and lower polariton (LP) branches of the coupled mode analysis are represented by the white dashed lines. In the inset, the A-exciton wavelength λ_A_ is represented by the dashed line (black) and the GMR dispersion by the dotted line (white). **f** Modulation depth as function of grating period, with the designed period *Λ* indicated (dashed line). The color scale is capped at ±12 dB for visibility

To start, we tailor the bottom hBN thickness such that the monolayer is positioned at a *λ*/2 separation from the back-reflector (Fig. 2a). In concept, this is reminiscent of the famous Salisbury screen, in which a thin absorber is separated *λ*/4 from a mirror to enhance absorption through constructive interference. In contrast, placing the WS_2_ at *λ*/2 instead minimizes absorption losses in the cavity due to destructive interference at the monolayer position. Since TM-polarized light cannot couple to the TE_0_ guided mode supported by the structure, it is predominantly reflected from the cavity in this spectral and angular range (Supplementary Fig. S1). On the other hand, TE-polarized light can pick up first-order grating momentum (equal to 2π/*Λ*, where *Λ* is the grating period) and thereby couple to the GMR, which enhances the interaction of light with the monolayer by confining the field inside the structure.

In our simulations, we phenomenologically capture the doping-dependent optical response of monolayer WS_2_ (Fig. 2b) by allowing the A-exciton nonradiative decay rate, *γ*_A,nr_, to increase with electron density. In the presence of excess charge carriers, excitons can convert to trions, which exhibit a comparatively weak oscillator strength and decay mostly nonradiatively^7,42^. Consequently, the trion does not appear as a distinct resonance in reflection (Supplementary Fig. S2), and we treat trion formation as an additional nonradiative decay channel for the exciton. Accordingly, we write *γ*_A,nr_ = *γ*_A,ph_ + *γ*_A,e_ + *γ*_A,T_, where *γ*_A,ph_ and *γ*_A,e_ denote the phonon- and electron-assisted nonradiative decay rates and *γ*_A,T_ corresponds to the exciton-to-trion conversion rate^30^. In the intrinsic doping regime, phonon-assisted decay processes dominate, and we set the nonradiative decay to occur ten times faster than the radiative decay (*γ*_A,r_), with characteristic values *ħγ*_A,r_ = 2.7 meV and *ħγ*_A,nr_ = 27 meV chosen to match the rates observed in preliminary experiments. At strong n-type doping, the exciton transition is severely broadened due to the increased exciton-electron scattering rate and the formation of trions^5–7^, which we model by artificially increasing the nonradiative decay rate to *ħγ*_A,nr_ = 270 meV. In this regime, absorptive losses in the cavity reduce to those suffered evanescently in the gold and by means of sub-bandgap absorption in monolayer WS_2_ owing to impurities, defects, and edge states^43^.

We optimize the grating periodicity such that the GMR coupling condition is satisfied at normal incidence for a photon energy matching the exciton energy, *E*_A_ = 2.00 eV, while the duty cycle is tailored to achieve perfect absorption when the monolayer is strongly n-doped (Fig. 2c). The resulting dispersion relation simply resembles the bare cavity dispersion with a slightly broader linewidth (Supplementary Fig. S3). Crucially, the n-doped dispersion is critically coupled such that the reflectance dips to *R*~0, and the Γ-point is located near *E*_A_. As a consequence, a gap opens in the dispersion around *E*_A_ when the monolayer is neutralized^44,45^, indicative of strong exciton-photon interactions (Fig. 2d).

To evaluate the coupling strength, we additionally simulate the normal-incidence reflectance as a function of grating period (Fig. 2e). This reveals the characteristic anti-crossing behavior associated with strong light-matter coupling, where two hybridized polariton modes emerge due to the rapid energy exchange between photons and a material resonance. The energy separation is proportional to the coupling strength $g=\sqrt{n}\mu E$, where *n* is the exciton density with transition dipole moment *μ*, and *E* is the local electric field strength^46^. At resonance, a pair of coupled oscillators is said to be strongly coupled if the mode splitting, characterized by the Rabi energy *Ω*_R_, is larger than the average decay rate of the uncoupled resonances, *i.e*., *Ω*_R_ > (*ħγ*_A_ + *ħγ*_GMR_)/2. From simulations of the cavity in the absence of the exciton resonance, we determine *ħγ*_GMR_ = 23.3 meV for the bare GMR, in good agreement with the experimentally estimated value of 22.9 meV. We subsequently fit the dispersion at intrinsic doping using a coupled mode theory (CMT) analysis^47,48^ (see Methods for details). From this, we extract a Rabi splitting of *Ω*_R_ = 38.2 meV and a corresponding coupling strength *ħg* = 19.1 meV, putting the system well into the regime of strong coupling (>25.2 meV). Instead, in the strongly n-doped regime (inset of Fig. 2e), the exciton decay rate is so large that *Ω*_R_ becomes purely imaginary and the gap closes, as expected for a weakly coupled system. Our metasurface leverages this tunability between strong and weak coupling to achieve >12 dB reflectance modulation in a broad wavelength range around *E*_A_ (Fig. 2f). This highlights how integration of a 2D semiconductor in a hybrid metasurface configuration considerably enhances the excitonic modulation depth.

### Reflectance modulation by gate-tuning of excitons

**Fig. 3:**
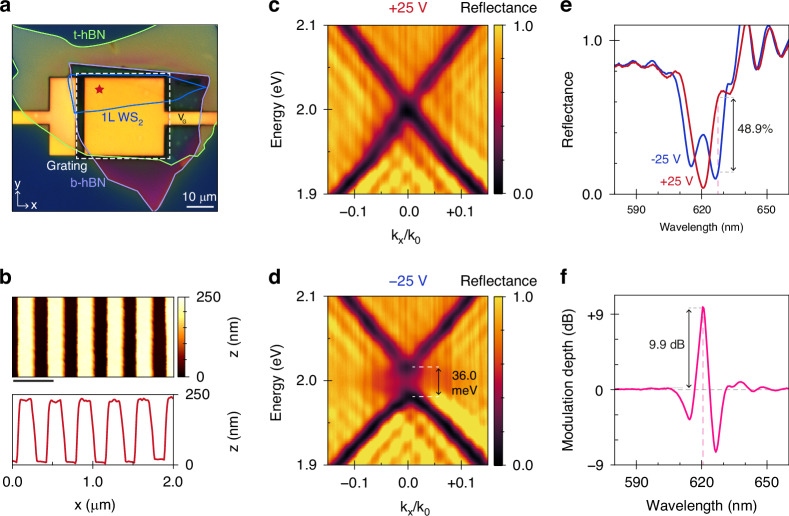
Free-space optical modulation via electrostatic gating of a hybrid-2D metasurface. **a** Optical microscope image of the hybrid-2D metasurface: a heterostructure comprised of monolayer WS_2_ (blue) encapsulated by bottom (purple) and top (green) hBN layers is placed on a gold back-reflector, which doubles as the gate electrode (V_G_), and integrated with a dielectric grating (white, dashed). The red star indicates the position of the **b** atomic force micrograph (top) and horizontal cross-section through the center (bottom). Scalebar: 500 nm. **c** Experimental back-focal plane imaging spectroscopy of the angular dispersion at strong n-type doping (+25 V, red) and **d** intrinsic doping (−25 V, blue), with the latter exhibiting a Rabi splitting energy of *Ω*_R_ = 36.0 meV. **e** Normal-incidence TE-polarized reflectance spectra extracted from (**c**) and (**d**) at *k*_x_/*k*_0_ = 0, with a maximum reflectance contrast of 48.9% at *λ*_0_ = 627.5 nm indicated. **f** Modulation depth obtained from the spectra in (**e**), revealing 9.9 dB peak signal modulation at *λ*_0_ = 620.5 nm

To demonstrate our approach experimentally, we employ a dry-transfer technique to assemble mechanically exfoliated WS_2_ and hBN flakes on prepatterned Au electrodes^49^. We functionalize the heterostructure cavity by nanopatterning a subwavelength grating in a spin-coated layer of CSAR (chemical semi-amplified resist) using electron-beam lithography (Fig. 3a). Atomic force microscopy confirms the grating’s periodicity and reveals excellent uniformity of the grating lines (Fig. 3b), while photoluminescence mapping spectroscopy (Supplementary Fig. S4) verifies that the exciton resonance is not detrimentally red-shifted or broadened during fabrication by virtue of the heterostructure encapsulation. We proceed by measuring the angle-resolved reflectance of the device via back-focal plane imaging spectroscopy (Supplementary Fig. S5). First, we apply a +25 V gate bias to induce strong n-doping of the monolayer and thereby quench the exciton resonance. As shown in Fig. 3c, the dispersion relation in this state resembles that of the bare cavity (Supplementary Fig. S6). Critically, the Γ-point is located very close to *E*_A_, such that when we neutralize the monolayer at –25 V, an energy gap of *Ω*_R_ = 36.0 meV opens in the dispersion due to strong exciton-photon coupling (Fig. 3d).

Before proceeding, we make two noteworthy observations about these results. First, to neutralize the WS_2_, we reverse the bias from +25 V (electron injection) to –25 V (hole injection), indicating that the monolayer is significantly n-doped in the unbiased state. This is rationalized by the natural n-doping of exfoliated transition metal dichalcogenides due to chalcogen atom vacancies as well as extrinsic disorder incurred during (nano)fabrication^50–52^. Despite this, the device performance is not significantly impaired because the effects of unwanted doping are readily mitigated via electrostatic gating. Second, the fact that the absolute reflectance exceeds unity does not imply gain in this specific scenario. On the one hand, we attribute this to a small normalization error caused by laser power fluctuations and a suboptimal choice of reflection reference in our experiments. On the other hand, we observe a distinct interference pattern in the dispersion diagrams arising from the finite lateral extent of the cavity (*i.e*., a finite-size effect). Due to the non-local character of the GMR, light is periodically re-radiated from the structure and interferes in the far-field. Analogous to Fraunhofer diffraction, the scattered amplitude is proportional to the Fourier transform of the near-field, given by a rectangular aperture convolved with the in-plane wavevector of the quasi-guided mode. This redistributes power across *k*-space and manifests as oscillations in reflectance surpassing unity. Yet, apart from these deviations, the measured dispersion relations are in excellent agreement with our numerical simulations (Fig. 2c, d).

Next, we extract cross sections from the dispersion relations at *k*_x_ = 0 μm^−^^1^ (Fig. 3e). At +25 V, the exciton is suppressed, and the reflectance spectrum contains one near-critically coupled resonance that dips to a minimum of 4% at the operating wavelength *λ*_0_ = 620.5 nm. Reversing the bias to –25 V neutralizes the monolayer, causing the spectrum to split into two strongly coupled polariton branches and thereby raising the reflectance to 39%. By switching between these two states, our modulator achieves a modulation depth of 9.9 dB (*ΔR* = 35%) at the operating wavelength (Fig. 3f) and a change in reflectance up to *ΔR* = 49% at 627.5 nm. To our knowledge, the measured modulation ratio and reflectance contrast are ~5 times greater than previously reported for excitonic metasurfaces at room temperature^30^, demonstrating the merits of our hybrid-2D approach.

We additionally assess the modulation frequency response of two more devices in alternating current experiments (Supplementary Fig. S7 and Fig. S5b) and measure a maximum –3 dB bandwidth of *f*_*-3dB*_ = 26 Hz. While, in principle, the solid-state gating mechanism of excitons allows for very rapid modulation, the low bandwidth observed here can be attributed to the large resistor-capacitor time constant of the electrical circuit owing to the high contact resistance between Au and WS_2_^53^. We emphasize this is merely a technological rather than a fundamental limitation, as bandwidths exceeding the megahertz regime have already been realized with optimized contact geometries in similar device architectures^29,36^.

### Electrically tunable strong coupling

**Fig. 4:**
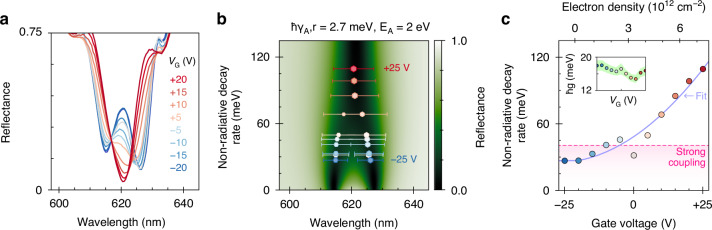
Tunable strong coupling via electrostatic gating. **a** Normal-incidence TE-polarized reflectance as function of gate voltage (color). The spectra measured at *V*_G_ = 0 V, ±25 V are omitted for clarity. **b** Spectral reflectance versus nonradiative decay rate of excitons in monolayer WS_2_, simulated with the RCWA model for *ħγ*_A,r_ = 2.7 meV and *E*_A_ = 2 eV. The colored datapoints represent the fitted energies, relative amplitudes (position and size of hexagons, respectively), and linewidths (width of bars) of the coupled modes. **c** Exciton nonradiative decay rate as a function of gate voltage (electron density) obtained from the RCWA fits (colored datapoints). Purple curve indicates the fitted optical rate equation, pink-shaded region represents the decay rates at which the system is strongly coupled. The coupling rates *ħg* obtained from the CMT analysis are shown in the inset (fitting uncertainty in green)

To gain insight into the voltage-dependence of the fundamental rates governing the metasurface’s optical response, we measure the normal-incidence reflectance at gate voltages between ±25 V in intervals of 5 V (Fig. 4a). With increasing voltage, the coupled modes gradually merge into a single resonance, continuously transitioning from strong to weak coupling^26–29,44^. In the following, we quantify the characteristic decay and coupling rates from our measurements by performing a comparative analysis using our RCWA and CMT models.

To accurately model the fabricated device in our RCWA simulations, we narrow down the exact geometry using reflectometry and atomic force microscopy, measure the relative permittivity of CSAR with spectroscopic ellipsometry, and obtain the dielectric functions of the other materials from literature^54–56^. For monolayer WS_2_, we model the permittivity with a Cauchy-Lorentz oscillator model where we assign an oscillator to each excitonic feature and a constant background term to account for higher-order resonances^4,23,56,57^ (see Methods for details). In our analysis, the only remaining fitting parameters are the A-exciton energy and decay rates. We start by fitting the measurements at intrinsic doping (Supplementary Fig. S8a, b) and obtain *E*_A_ = 2.00 eV, *ħγ*_A,r_ = 2.7 meV and *ħγ*_A,nr_ = 26.7 meV, closely matching the values used for our numerical simulations (Fig. 2). In the following, we assume that *γ*_A,r_ is voltage-independent, since it would otherwise over-parametrize the model as *γ*_A,r_/*γ*_A,nr_ → 0, while noting that this may be a slight oversimplification^4^. Nevertheless, the modulator’s voltage-dependent optical response is well-captured by the RCWA model (Fig. 4b), with the simulated reflectance map clearly showing two distinct exciton-polariton branches that gradually merge into a single resonance with increasing *γ*_A,nr_. As such, we continue the fitting procedure to extract *γ*_A,nr_ (and *E*_A_) at each voltage (Fig. 4c). We find that, in the strongly n-doped regime (at +25 V), the decay rate is increased fourfold to *ħγ*_A,nr_ = 109.4 meV while the resonance energy is redshifted to *E*_A_ = 1.99 eV. We emphasize that the modulator’s performance could be improved even further by increasing *γ*_A,nr_ to achieve true critical coupling. The observed redshift is attributed to a doping-induced lowering of the exciton binding energy due to screening of the Coulomb interaction, consistent with earlier work^6^. Regardless, the data is captured exceptionally well by our model, allowing us to directly overlay the fitting results on the simulated reflectance map (Fig. 4b) and confirming the validity of our approach.

Next, we determine the exciton-photon coupling strength (inset of Fig. 4c) by plugging the extracted exciton parameters into our CMT model and fitting it to the experimental data (Supplementary Fig. S8c, d). From this, we estimate a voltage-averaged value of *ħg* = 16.6 ± 1.0 meV and find that the excitonic and photonic modes remain strongly coupled up to a total exciton decay rate of *γ*_A,max_ = 43.3 meV. Notably, the measurement at 0 V is also strongly coupled and appears to be a significant outlier. However, this is explained by hysteresis due to trapped charges^50^ resulting from an imperfect measurement sequence in which the monolayer was slightly p-doped (at −25 V) before sweeping the gate voltage from 0 to +25 V and finally from –5 back to –25 V. Besides, we note that the trion resonance is excluded in this CMT analysis, because it is only weakly coupled to the GMR. As such, it negligibly impacts the fitted reflectance spectra or the extracted exciton-photon coupling strength (Supplementary Note 1).

To gain insight in the gating-induced doping density *n*_e_(*V*_G_) in the WS_2_ monolayer, we describe our device as a parallel-plate capacitor modified to account for quantum capacitance effects^58,59^. We fit the experimental decay rates by plugging the expression for *n*_e_ into the optical rate-equation model proposed in earlier work^30,42^. From the fit, we obtain an estimate of the charge neutrality point, *V*_CNP_ = − 21.3 V, which enables us to calibrate *n*_e_ with respect to *V*_G_ (Fig. 4c). We estimate carrier concentrations ranging from *n*_e_ ≈ −0.4⋅10^12^ cm^−^^2^ at −25 V up to *n*_e_ ≈ 7.3⋅10^12^ cm^−2^ at +25 V, with the maximum electron density up to which the device remains strongly coupled *n*_e,max_ ≈ 2.4⋅10^12^ cm^−2^ (−5.2 V). Altogether, our results reveal that the metasurface’s ability to electrically tune the hybrid-2D system in and out of the strong exciton-photon coupling regime fundamentally stems from doping-induced variations in the nonradiative decay rate of excitons in monolayer WS_2_.

## Discussion

This work introduces a hybrid-2D metasurface platform that combines the merits of a tunable Van der Waals heterostructure cavity with the strong light-matter interaction of a non-local dielectric metasurface to achieve strong and tunable exciton-photon interactions at room temperature, which is leveraged for free-space optical modulation. The platform’s simple yet versatile design readily accommodates other materials and is straightforwardly adapted to polarization-independent response and various optical functionalities. More broadly, our platform highlights new opportunities for excitonic 2D metasurfaces for dynamic wavefront manipulation in ultra-compact metadevices.

## Materials and methods

### Sample fabrication

#### Lithography of pre-patterned substrate

We prepattern Au contacts on diced 12 × 12 mm^2^ Si substrates with 100 nm dry-grown thermal oxide (University Wafer). For this, we spin-coat a 400 nm layer of positive-tone electron-beam (e-beam) resist CSAR AR-P 6200 (AllResist GmbH) using a Süss MicroTec Delta 80 and cure it at 175 °C for 120 seconds. We pattern the contact outlines in the resist using e-beam lithography (Raith Voyager, 50 kV acceleration voltage) with a dose of 152.2 μC/cm², and develop them by soaking for 60 seconds in n-amyl acetate, followed by 7 seconds in o-xylene and 15 seconds in MIBK:IPA 9:1. After development, we rinse the substrates in IPA and blow-dry them with N_2_ gas. With the mask completed, we deposit a 3 nm adhesion layer of Cr and 100 nm Au using a custom-built thermal evaporator. We perform lift-off by soaking the samples in methoxybenzene (anisole) at 70 °C for 20 minutes. Subsequently, we clean the substrates thoroughly with a base piranha solution (1:1 vol. NH_3_:H_2_O_2_) at 75 °C for 10 minutes, rinse briefly in IPA, and blow-dry with N_2_ gas. Finally, we treat the substrates in O_2_/N_2_ plasma for 2 minutes at 70 W (Diener Zepto) to complete the prepatterned substrates.

#### Heterostructure fabrication

We obtain hBN and WS_2_ bulk crystals commercially (HQ Graphene) and manually exfoliate them using Nitto SPV-224 tape. We transfer the exfoliated flakes onto stamps cut from polydimethylsiloxane sheets (Gel-Pak WF-30). Using a confocal WITec α-300 microscope, we identify hBN flakes of suitable thickness with white-light reflectometry and WS_2_ monolayers with direct photoluminescence imaging using a 405 nm widefield excitation source (Becker & Hickl) and a 550 nm long-pass filter (Thorlabs). After identifying the candidate flakes, we mount a prepatterned substrate in a custom stamping microscope with a heating stage. We align the bottom hBN flake with the back-reflector (gate) and the ground electrode, then bring it into contact. We heat the stack to 60 °C and thermalize it for at least 5 minutes. Subsequently, we peel the stamp slowly from the substrate at 0.1 μm/s to transfer the hBN flake. We thermally anneal the substrate with the hBN in a tube furnace in vacuum (*P* ≈ 10^−6^ – 10^−7 ^mbar) at 150 °C for 8 hours to promote adhesion and clean the exposed surface. We repeat this process for the WS_2_ monolayer (annealing at 90 °C for 8 hours) and the top hBN layer (120 °C for 24 hours), completing the heterostructure cavity.

#### Lithography of subwavelength grating

To fabricate the subwavelength gratings, we clean the devices briefly with a 30 second O_2_ descum (Oxford Instruments Plasma 80). We spin-coat a ~220 nm thick layer of CSAR AR-P 62 e-beam resist and cure it at 150 °C for 120 seconds. Using the e-beam lithography system (Raith Voyager), we write the grating patterns with an electron dose of 130.8 μC/cm^2^. We develop the gratings by soaking the samples for 60 seconds in n-amyl acetate, followed by 7 seconds in o-xylene and 15 seconds in MIBK:IPA 9:1. We rinse the samples in IPA and dry them with N_2_ gas.

#### Electrical connections

To enable electrical contact and mounting in the optical microscope, we attach one of the samples to a custom printed circuit board (PCB) using double-sided tape. We wire-bond the gate and ground electrodes to the PCB with 25 μm diameter Al wires (West.Bond 7KE), connecting them to two separate pads on the PCB.

### Optical measurements

#### Back-focal plane reflection

We perform in-situ back-focal plane (BFP) reflection measurements with a WITec α300 confocal microscope and an NKT Photonics SuperK laser system as the light source (Supplementary Fig. S4a). We generate linearly polarized monochromatic light (~1 nm bandwidth) with a pulsed (78 MHz repetition rate, ~5 ps pulse width) supercontinuum white-light laser (Extreme EXW-12) and an acousto-optically tunable filter (Select VIS/1X), feeding it into the microscope via a single-mode photonic crystal fiber (Connect FD7-PM). The sample is mounted on the microscope’s piezo stage and illuminated with a 20x objective (Zeiss EC Epiplan, NA = 0.4) that is defocused by 5 μm. The incident power is varied per wavelength to maximize the signal-to-noise ratio and is in the range of *P* = 36–80 nW (fluence ranging from *F* = 7–16 nJ/cm^2^). To switch between TE and TM polarized illumination, we rotate the sample (holder) by a quarter turn. We capture the reflected light with the same objective, pass it through a polarization analyzer and the Bertrand lens, and project the BFP onto a 14-bit CMOS camera (Zeiss Axiocam 705 mono). We sweep the laser across the spectrum in steps of 0.5 nm or 1 nm and record an image of the Fourier plane at each wavelength, adjusting the camera’s exposure time to account for spectral variation in laser power. For each measurement (*I*_sample_), we record two reference measurements: one without a sample mounted to remove dark counts and internal reflections (*I*_dark_), and another on an exposed gold electrode (*I*_reference_) to normalize the data using the theoretical angle-resolved reflectance of 100 nm gold (*R*_reference_), simulated with RCWA (Supplementary Fig. S9). The absolute reflectance is then calculated as:

$$R=\frac{{I}_{\mathrm{sample}}-\,{I}_{\mathrm{dark}}}{{I}_{\mathrm{reference}}-\,{I}_{\mathrm{dark}}}\,\cdot \,{R}_{\mathrm{reference}}$$

For DC gating measurements, we apply a gate bias with a Keithley 2612B SourceMeter, sweeping from 0 to +25 V and then from −5 to −25 V in 5 V steps. We use custom Python scripts to interface with the equipment and automate the experiment.

We extract the *k*_x_ and *k*_y_ dispersions from the BFP images by determining the BFP center (*k*_x_ = *k*_y_ = 0 μm^−1^) manually and defining the coordinate transformation between image pixels and the lens’s NA using the known BFP radius (in pixels). A distinct dust particle visible on all BFP images serves to align the images, ensuring a common center coordinate. We define the *k*_x_ = 0 and *k*_y_ = 0 μm^−1^ lines based on the sample’s orientation and enhance the signal-to-noise ratio by integrating traces over a small angular region (−3 ≤ θ ≤ 3 mrad, 11 pixels). By repeating this process at each wavelength, we construct line-by-line images of the BFP dispersion to generate the diagrams shown in (Fig. 3c, d). We extract the normal-incidence reflectance spectra (Figs. 3e, 4a) and modulation depth (Fig. 3f) from these diagrams by taking wavelength (energy) traces at *k*_x_ = 0 μm^−1^.

#### Alternating current modulation

We perform alternating current measurements (Supplementary Fig. S7) using a modified optical microscope setup for BFP measurements (Supplementary Fig. S5b). We feed the tunable laser (NKT) into the microscope (WITec) at another fiber port, adding a lens to achieve collimated wide-field excitation after passing through the 20x objective lens (Zeiss). An Agilent HP 33120 A function generator provides the square-wave modulation signal. The modulated beam is collected with the same objective, outcoupled into a fiber, and detected with a biased Si photodiode (Thorlabs DET100A2). We connect the photodiode electrically to a source-measure unit (Keithley) and amplify the photogenerated current (responsivity ~0.43 A/W) while converting it to voltage (0.5 nA/V) with the Stanford Research Systems (SRS) SR-570 transimpedance amplifier. Finally, we measure the signal using an SR-830 lock-in amplifier (SRS) or a TDS 3032 oscilloscope (Tektronix) synced to the function generator. All measurements are automated using custom Python scripts.

#### Spectrally resolved photoluminescence mapping

We perform photoluminescence (PL) spectroscopy and mapping in the WITec microscope with a 100x objective (Zeiss EC Epiplan-Neofluar, NA = 0.9) in dark-field mode. We use a fiber-coupled 532 nm diode laser to excite a diffraction-limited spot on the sample. The collected PL emission is chromatically dispersed by a WITec UHTS300 SMFC VIS spectrograph (600 lines/mm), and we record the spectra on an Andor Newton EMCCD camera cooled to −60 °C. For spatial mapping, we precisely control the sample position using a piezo-actuated stage and acquire spectra on a rectangular grid with 333 nm spacing.

### Numerical simulations

#### Rigorous coupled-wave analysis

Numerical simulations are performed using the S^4^ RCWA package available for Python^60^. We obtain the refractive indices of Au and hBN from literature as tabulated data^54,55^, while for CSAR, we measure it through spectroscopic ellipsometry (J.A. Woollam VB-400) of the material spin-coated on a Si substrate. For monolayer WS_2_, we use the Cauchy-Lorentz oscillator model to express the permittivity as a set of excitonic oscillators—each characterized by a resonance energy, oscillator strength, and total decay rate—plus a constant background term to account for higher-order resonances^4,23,56,57^. We modify the model by expressing the A-exciton’s oscillator strength in terms of its radiative decay rate *γ*_A,r_ as *f*_A_ ≈ *ħγ*_A,r_ ⋅ 2*ħcd*^*-1*^, where *ħ* is the reduced Planck’s constant, *c* the speed of light, and *d* = 6.18 Å the thickness of the monolayer WS_2_^61^. To start, the A-exciton energy and linewidth are estimated from preliminary measurements and checked by fitting a spectrally resolved PL map (Supplementary Fig. S4). We narrow down the exact geometry of the fabricated modulator by measuring the thicknesses of the hBN layers using white-light reflectometry in the WITec microscope, and the period, height, and width of the CSAR grating using atomic force microscopy (Bruker Dimension FastScan, Fig. 3b). To fit the voltage-dependent reflectance spectra, we initially treat the A-exciton and the geometry as fitting parameters constrained by the experimental uncertainty to account for variations across the sample area. We start by fitting the reflectance at *V*_*G*_ = − 25 V because the clearly distinguishable polariton branches allow for reliable determination of the desired parameters (Supplementary Fig. S8a). In subsequent fits, the geometry is fixed because it does not change with voltage, and *γ*_A,r_ is fixed because it would over-parametrize the model in the exciton-quenched regime.

#### Coupled mode theory

We employ a CMT analysis to evaluate the strong coupling condition and to extract the coupling strength *g* and polariton dispersions. We define the complex energy of oscillator *j* as ${\widetilde{E}}_{\text{j}}={E}_{\text{j}}-i\hslash {\varGamma }_{\text{j}}$, with resonance energy *E*_j_ and damping rate ${\varGamma }_{\text{j}}=\gamma /2$, such that the eigenenergies of the coupled excitonic (A) and photonic (GMR) modes are given by:

$${\widetilde{E}}_{\pm }=\left(\tfrac{{\widetilde{E}}_{\text{GMR}}+{\widetilde{E}}_{\text{A}}}{2}\right)\pm \sqrt{{(\hslash g)}^{2}-{\left(\tfrac{i({\widetilde{E}}_{\text{GMR}}-{\widetilde{E}}_{\text{A}})}{2}\right)}^{2}}$$

The Rabi splitting is defined as ${\varOmega }_{\text{R}}={\widetilde{E}}_{+}-{\widetilde{E}}_{-}$ and the strong coupling criterion as ${\mathfrak{R}}\left({\varOmega }_{\text{R}}\right) > {\hslash \varGamma }_{\text{GMR}}+\hslash {\varGamma }_{\text{A}}$. In evaluating this condition, we account for doping-dependent variations in *E*_A_ by invoking *E*_GMR_ = *E*_A_ such that the Rabi splitting energy reduces to:

$${\varOmega }_{{\rm{R}}}=\hslash \sqrt{4{g}^{2}-{\left({\varGamma }_{\text{GMR}}-{\varGamma }_{\text{A}}\right)}^{2}}$$

To fit the experimental reflectance (Supplementary Fig. S8c, d), we model the non-resonant background reflection using RCWA simulations for TM-polarized illumination (Fig. 2b). We plug the extracted values of ${\widetilde{E}}_{\text{A}}$ and ${\widetilde{E}}_{\text{GMR}}$ from our RCWA fits (Supplementary Fig. S8a, b) into the CMT model, leaving only *g* and two normalization terms *I*_A_ and *I*_GMR_ as free fitting parameters, which we obtain via a least-squares fitting routine. The uncertainty in *g* is quantified as the mean relative error between data and fit (inset of Fig. 4c). The numerical analyses are performed using custom Python scripts.

#### Carrier density calculations

For calculations of the 2D electron density *n*_e_ as a function of the applied gate bias, we model our device as a parallel-plate capacitor. We modify the well-known expression for the parallel-plate capacitance, *C*_geo_ = *ε*_0_*ε*_hBN_/*d*_hBN_, to account for the quantum capacitance^58,59^, *C*_q_ = *e*^2^*g*_s_*g*_v_m*/(*πħ*^2^), such that the overall capacitance per unit area is given as *C* = (1/*C*_geo_ + 1/*C*_q_)^-1^. We use a custom Python script to calculate *n*_e_ by plugging the measured hBN thickness *d*_hBN_ = 117 nm and its static permittivity^62^ ε_hBN_ ≈ 3.4, together with values for the monolayer WS_2_ bandgap energy^63^ *E*_bg_ ≈ 2.15 eV, valley degeneracy *g*_v_ = 2, spin degeneracy *g*_s_ = 2, and effective electron mass^64^ m* ≈ 0.34*m*_*e*_. We obtain an estimate of the charge neutrality point by fitting *γ*_A,nr_ (*n*_e_) using an optical rate-equation model^30,42^ (Fig. 4c).

## Supplementary information

Supporting materials

## Data Availability

A full replication package, including all data and scripts, is openly available at 10.6084/m9.figshare.30051241.

## References

[1] Geim, A. K. & Grigorieva, I. V. Van der Waals heterostructures. *Nature* **499**, 419–425 (2013). 23887427 10.1038/nature12385

[2] Novoselov, K. S. et al. Electric field effect in atomically thin carbon films. *Science* **306**, 666–669 (2004). 15499015 10.1126/science.1102896

[3] Chernikov, A. et al. Exciton binding energy and nonhydrogenic Rydberg series in monolayer WS_2_. *Phys. Rev. Lett.* **113**, 076802 (2014). 25170725 10.1103/PhysRevLett.113.076802

[4] Li, M. et al. Refractive index modulation in monolayer molybdenum diselenide. *Nano Lett.* **21**, 7602–7608 (2021). 34468150 10.1021/acs.nanolett.1c02199

[5] Yu, Y. L. et al. Giant gating tunability of optical refractive index in transition metal dichalcogenide monolayers. *Nano Lett.* **17**, 3613–3618 (2017). 28505462 10.1021/acs.nanolett.7b00768

[6] Chernikov, A. et al. Electrical tuning of exciton binding energies in monolayer WS_2_. *Phys. Rev. Lett.* **115**, 126802 (2015). 26431003 10.1103/PhysRevLett.115.126802

[7] Ross, J. S. et al. Electrical control of neutral and charged excitons in a monolayer semiconductor. *Nat. Commun.* **4**, 1474 (2013). 23403575 10.1038/ncomms2498

[8] Zhou, Y. et al. Controlling excitons in an atomically thin membrane with a mirror. *Phys. Rev. Lett.* **124**, 027401 (2020). 32004011 10.1103/PhysRevLett.124.027401

[9] Kim, I. et al. Nanophotonics for light detection and ranging technology. *Nat. Nanotechnol.* **16**, 508–524 (2021). 33958762 10.1038/s41565-021-00895-3

[10] Miller, D. A. B. Attojoule optoelectronics for low-energy information processing and communications. *J. Lightw. Technol.* **35**, 346–396 (2017).

[11] Zheludev, N. I. & Kivshar, Y. S. From metamaterials to metadevices. *Nat. Mater.* **11**, 917–924 (2012). 23089997 10.1038/nmat3431

[12] Kamali, S. M. et al. A review of dielectric optical metasurfaces for wavefront control. *Nanophotonics* **7**, 1041–1068 (2018).

[13] Shaltout, A. M., Shalaev, V. M. & Brongersma, M. L. Spatiotemporal light control with active metasurfaces. *Science* **364**, eaat3100 (2019). 31097638 10.1126/science.aat3100

[14] Overvig, A. & Alù, A. Diffractive nonlocal metasurfaces. *Laser Photonics Rev.* **16**, 2100633 (2022).

[15] Gu, T. et al. Reconfigurable metasurfaces towards commercial success. *Nat. Photonics* **17**, 48–58 (2023).

[16] Kuznetsov, A. I. et al. Roadmap for optical metasurfaces. *ACS Photonics* **11**, 816–865 (2024). 38550347 10.1021/acsphotonics.3c00457 PMC10971570

[17] Shirmanesh, G. K. et al. Electro-optically tunable multifunctional metasurfaces. *ACS Nano* **14**, 6912–6920 (2020). 32352740 10.1021/acsnano.0c01269

[18] Benea-Chelmus, I. C. et al. Gigahertz free-space electro-optic modulators based on Mie resonances. *Nat. Commun.* **13**, 3170 (2022). 35668071 10.1038/s41467-022-30451-z PMC9170732

[19] Sherrott, M. C. et al. Experimental demonstration of >230° phase modulation in gate-tunable graphene-gold reconfigurable mid-infrared metasurfaces. *Nano Lett.* **17**, 3027–3034 (2017). 28445068 10.1021/acs.nanolett.7b00359

[20] Holsteen, A. L. et al. Purcell effect for active tuning of light scattering from semiconductor optical antennas. *Science* **358**, 1407–1410 (2017). 29242341 10.1126/science.aao5371

[21] Pitanti, A. et al. Gigahertz modulation of a fully dielectric nonlocal metasurface. *Adv. Opt. Mater.* **12**, 2401283 (2024).

[22] van de Groep, J. et al. Exciton resonance tuning of an atomically thin lens. *Nat. Photonics* **14**, 426–430 (2020).

[23] Guarneri, L. et al. Temperature-dependent excitonic light manipulation with atomically thin optical elements. *Nano Lett.* **24**, 6240–6246 (2024). 38578061 10.1021/acs.nanolett.4c00694 PMC11140734

[24] Epstein, I. et al. Near-unity light absorption in a monolayer WS_2_ van der Waals heterostructure cavity. *Nano Lett.* **20**, 3545–3552 (2020). 32283034 10.1021/acs.nanolett.0c00492

[25] Li, B. W. et al. Single-nanoparticle plasmonic electro-optic modulator based on MoS_2_ monolayers. *ACS Nano* **11**, 9720–9727 (2017). 28863263 10.1021/acsnano.7b05479

[26] Lee, B. et al. Electrical tuning of exciton–plasmon polariton coupling in monolayer MoS_2_ integrated with plasmonic nanoantenna lattice. *Nano Lett.* **17**, 4541–4547 (2017). 28613887 10.1021/acs.nanolett.7b02245

[27] Zhang, L. et al. Photonic-crystal exciton-polaritons in monolayer semiconductors. *Nat. Commun.* **9**, 713 (2018). 29459736 10.1038/s41467-018-03188-x PMC5818602

[28] Fernandez, H. A. et al. Electrically tuneable exciton-polaritons through free electron doping in monolayer WS_2_ microcavities. *Adv. Opt. Mater.* **7**, 1900484 (2019).

[29] Dibos, A. M. et al. Electrically tunable exciton–plasmon coupling in a WSe_2_ monolayer embedded in a plasmonic crystal cavity. *Nano Lett.* **19**, 3543–3547 (2019). 31117747 10.1021/acs.nanolett.9b00484

[30] Li, Q. T. et al. A purcell-enabled monolayer semiconductor free-space optical modulator. *Nat. Photonics* **17**, 897–903 (2023).

[31] Song, J. G. et al. Effect of Al_2_O_3_ deposition on performance of top-gated monolayer MoS_2_-based field effect transistor. *ACS Appl. Mater. Interfaces* **8**, 28130–28135 (2016). 27681666 10.1021/acsami.6b07271

[32] Moody, G. et al. Intrinsic homogeneous linewidth and broadening mechanisms of excitons in monolayer transition metal dichalcogenides. *Nat. Commun.* **6**, 8315 (2015). 26382305 10.1038/ncomms9315 PMC4595717

[33] Cadiz, F. et al. Excitonic linewidth approaching the homogeneous limit in MoS_2_-based van der Waals heterostructures. *Phys. Rev. X* **7**, 021026 (2017).

[34] Back, P. et al. Realization of an electrically tunable narrow-bandwidth atomically thin mirror using monolayer MoSe_2_. *Phys. Rev. Lett.* **120**, 037401 (2018). 29400509 10.1103/PhysRevLett.120.037401

[35] Scuri, G. et al. Large Excitonic reflectivity of monolayer MoSe_2_ encapsulated in hexagonal boron nitride. *Phys. Rev. Lett.* **120**, 037402 (2018). 29400519 10.1103/PhysRevLett.120.037402

[36] Andersen, T. I. et al. Beam steering at the nanosecond time scale with an atomically thin reflector. *Nat. Commun.* **13**, 3431 (2022). 35701395 10.1038/s41467-022-29976-0 PMC9198240

[37] Li, M. et al. Excitonic beam steering in an active van der Waals metasurface. *Nano Lett.* **23**, 2771–2777 (2023). 36921321 10.1021/acs.nanolett.3c00032

[38] Wang, Z. et al. Control over cavity exciton polaritons in monolayer semiconductors. Print at https://arxiv.org/abs/2311.03750 (2023).

[39] Sortino, L. et al. Atomic-layer assembly of ultrathin optical cavities in van der Waals heterostructure metasurfaces. *Nat. Photonics* **19**, 825–832 (2025).

[40] Wang, S. S. & Magnusson, R. Theory and applications of guided-mode resonance filters. *Appl. Opt.* **32**, 2606 (1993). 20820422 10.1364/AO.32.002606

[41] Mak, K. F. et al. Tightly bound trions in monolayer MoS_2_. *Nat. Mater.* **12**, 207–211 (2013). 23202371 10.1038/nmat3505

[42] Lien, D. H. et al. Electrical suppression of all nonradiative recombination pathways in monolayer semiconductors. *Science* **364**, 468–471 (2019). 31048488 10.1126/science.aaw8053

[43] Das, S. et al. Ultrafast transient sub-bandgap absorption of monolayer MoS_2_. *Light Sci. Appl.* **10**, 27 (2021). 33514690 10.1038/s41377-021-00462-4 PMC7846580

[44] Chakraborty, B. et al. Control of strong light–matter interaction in monolayer WS_2_ through electric field gating. *Nano Lett.* **18**, 6455–6460 (2018). 30160968 10.1021/acs.nanolett.8b02932

[45] Liu, X. Z. et al. Strong light–matter coupling in two-dimensional atomic crystals. *Nat. Photonics* **9**, 30–34 (2015).

[46] Dovzhenko, D. S. et al. Light-matter interaction in the strong coupling regime: configurations, conditions, and applications. *Nanoscale* **10**, 3589–3605 (2018). 29419830 10.1039/c7nr06917k

[47] Fan, S. H., Suh, W. & Joannopoulos, J. D. Temporal coupled-mode theory for the Fano resonance in optical resonators. *J. Opt. Soc. Am. A* **20**, 569 (2003). 10.1364/josaa.20.000569 12630843

[48] Yu, Y. L. & Cao, L. Y. Coupled leaky mode theory for light absorption in 2D, 1D, and 0D semiconductor nanostructures. *Opt. Express* **20**, 13847–13856 (2012). 22714450 10.1364/OE.20.013847

[49] Castellanos-Gomez, A. et al. Deterministic transfer of two-dimensional materials by all-dry viscoelastic stamping. *2D Mater.* **1**, 011002 (2014).

[50] Mitta, S. B. et al. Electrical characterization of 2D materials-based field-effect transistors. *2D Mater.* **8**, 012002 (2021).

[51] Rhodes, D. et al. Disorder in van der Waals heterostructures of 2D materials. *Nat. Mater.* **18**, 541–549 (2019). 31114069 10.1038/s41563-019-0366-8

[52] Daus, A. et al. High-performance flexible nanoscale transistors based on transition metal dichalcogenides. *Nat. Electron.* **4**, 495–501 (2021).

[53] Allain, A. et al. Electrical contacts to two-dimensional semiconductors. *Nat. Mater.* **14**, 1195–1205 (2015). 26585088 10.1038/nmat4452

[54] Yakubovsky, D. I. et al. Optical constants and structural properties of thin gold films. *Opt. Express* **25**, 25574–25587 (2017). 29041223 10.1364/OE.25.025574

[55] Lee, S. Y. et al. Refractive index dispersion of hexagonal boron nitride in the visible and near-infrared. *Phys. Status Solidi (B)* **256**, 1800417 (2019).

[56] Li, Y. L. et al. Measurement of the optical dielectric function of monolayer transition-metal dichalcogenides: MoS_2_, MoSe_2_, WS_2_, and WSe_2_. *Phys. Rev. B* **90**, 205422 (2014).

[57] Hsu, C. et al. Thickness-dependent refractive index of 1L, 2L, and 3L MoS_2_, MoSe_2_, WS_2_, and WSe_2_. *Adv. Opt. Mater.* **7**, 1900239 (2019).

[58] Brumme, T., Calandra, M. & Mauri, F. First-principles theory of field-effect doping in transition-metal dichalcogenides: structural properties, electronic structure, Hall coefficient, and electrical conductivity. *Phys. Rev. B* **91**, 155436 (2015).

[59] Ma, N. & Jena, D. Carrier statistics and quantum capacitance effects on mobility extraction in two-dimensional crystal semiconductor field-effect transistors. *2D Mater.* **2**, 015003 (2015).

[60] Liu, V. & Fan, S. H. S^4^: a free electromagnetic solver for layered periodic structures. *Comput. Phys. Commun.* **183**, 2233–2244 (2012).

[61] Wilson, J. A. & Yoffe, A. D. The transition metal dichalcogenides discussion and interpretation of the observed optical, electrical and structural properties. *Adv. Phys.* **18**, 193–335 (1969).

[62] Pierret, A. et al. Dielectric permittivity, conductivity and breakdown field of hexagonal boron nitride. *Mater. Res. Express* **9**, 065901 (2022).

[63] Roy, S. & Bermel, P. Electronic and optical properties of ultra-thin 2D tungsten disulfide for photovoltaic applications. *Sol. Energy Mater. Sol. Cells* **174**, 370–379 (2018).

[64] Liu, L. T. et al. Performance limits of monolayer transition metal dichalcogenide transistors. *IEEE Trans. Electron. Devices* **58**, 3042–3047 (2011).

