# Supplementary material for: Electrically tunable strong coupling in a hybrid-2D excitonic metasurface for optical modulation
Source: Light Sci Appl. 2026 Jan 2;15:28. doi: 10.1038/s41377-025-02079-3 (PMC12757595; doi:10.1038/s41377-025-02079-3)
Supplement: Supplementary file 1 — Supporting materials [file 41377_2025_2079_MOESM1_ESM.pdf]

Supporting Information for Electrically tunable strong coupling in a hybrid-2D excitonic metasurface for optical modulation

Tom Hoekstra[1] and Jorik van de Groep[1*]

[1] Van der Waals-Zeeman Institute, Institute of Physics, University of Amsterdam, 1098 XH, Amsterdam, the Netherlands

[*] j.vandegroep@uva.nl

Supporting Information

This file includes:

- Supplementary Note 1
- Supplementary Figures 1-10

Supplementary Note 1. Three-oscillator coupled-mode theory analysis of the optical coupling with trions.

To investigate the impact of optical coupling between trions (charged excitons) and guided photons on the exciton-photon coupling strength and the reflectivity of our metasurface, we additionally model the system using a coupled mode theory (CMT) with three oscillators representing the guided-mode, exciton and trion resonances (denoted with subscript c, x, and t, respectively)[1,2]. Since this is a one-port system and the coupling from free-space directly to excitons and trions is negligible due to destructive interference of free-space radiation at the monolayer position (Fig. 2a), the coupled equations in the steady-state are written as:

$$\frac{d}{dt}\begin{pmatrix} a_\mathrm{c} \\ a_x \\ a_t \end{pmatrix} = \begin{pmatrix} -i(\omega-\omega_c)+\Gamma_\mathrm{c} & -ig_x & -ig_t \\ -ig_x & -i(\omega-\omega_x)+\Gamma_x & 0 \\ -ig_t & 0 & -i(\omega-\omega_t)+\Gamma_t \end{pmatrix}\begin{pmatrix} a_\mathrm{c} \\ a_x \\ a_t \end{pmatrix} + \begin{pmatrix} \sqrt{2\Gamma_\mathrm{c,rad}} \\ 0 \\ 0 \end{pmatrix} S_\mathrm{in}$$

Here, a, ω, Γ, g represent the mode amplitude, resonance energy, damping rate (where subscript 'rad' denotes the radiative component) and coupling strength, respectively, and S_in is the free-space input field. By solving the set of coupled equations, the reflected signal is obtained from the input-output relation of the one-port system,

$$S_\mathrm{out} = S_\mathrm{in} - \sqrt{2\Gamma_\mathrm{c,rad}}\,a_c$$

The reflection coefficient is defined as $r = S_\mathrm{out}/S_\mathrm{in}$, thus yielding:

$$r = 1 - \frac{\sqrt{2\Gamma_\mathrm{c,rad}}\,a_c}{S_{in}}$$

To account for a slowly varying Fabry-Pérot interference component, we also include a frequency-dependent background term $r_\mathrm{bg}(\omega)$. Thus, we obtain the expression for the reflection coefficient:

$$r(\omega) = r_{bg}(\omega) - \frac{2\Gamma_\mathrm{c,rad}}{-i(\omega-\omega_c)+\Gamma_\mathrm{c} + \frac{g_x^2}{-i(\omega-\omega_x)+\Gamma_x} + \frac{g_t^2}{-i(\omega-\omega_t)+\Gamma_t}}$$

To analyze the impact of trions on $r(\omega)$ and g_x, we first fit the model in absence of trions (*i.e.* the two-oscillator CMT model) by setting the trion-photon coupling strength, $\hbar g_\mathrm{t} = 0$ meV. Plugging all the extracted values from our RCWA fits (described in the manuscript, see also Supplementary Fig. S8a, b) into the model leaves only g_x and $\Gamma_\mathrm{c,rad}$ as free parameters. Using a least-squares fitting routine, we fit the optical response at all voltages (–25 to +25 V in 5 V steps) and find $\hbar g_x = 20.98$ meV and $\hbar\Gamma_\mathrm{c,rad} = 7.45$ meV (Supplementary Fig. S10).

Next, we reintroduce the trion resonance with a characteristic energy $\omega_\mathrm{t} = 1.9630$ eV and a total decay rate of $\Gamma_t = 23.6$ meV obtained from the photoluminescence data (Supplementary Fig. S4). To evaluate the impact of trions coupling to the guided photons, we need to estimate the coupling strength g_t, which is proportional to the transition dipole moment μ_t and the local electric field strength E at the trion energy ω_t. Since the transition dipole moment is proportional to the square root of the oscillator strength $\mu_t \propto \sqrt{f_t}$, we can write $g_t \propto \sqrt{f_t}E(\omega_t)$ and similarly for the exciton resonance: $g_x \propto \sqrt{f_x}E(\omega_x)$. We may use this to estimate the trion-photon coupling strength:

$$\frac{g_t}{g_x} \propto \sqrt{\frac{f_t}{f_x}} \cdot \frac{E(\omega_t)}{E(\omega_x)}$$

$$g_t \approx g_x \cdot \sqrt{\frac{f_t}{f_x}} \cdot \frac{E(\omega_t)}{E(\omega_x)}$$

Lien *et al.*[3] show that in WS_2 the trion's oscillator strength is 16x smaller than the neutral exciton, such that $\sqrt{\frac{f_t}{f_x}} = 1/4$. Furthermore, we know, from *e.g.* Novotny & Hecht[4], that the frequency-dependent local electric field strength of the guided mode resonance can be expressed using a Lorentzian as:

$$|E(\omega)| \propto \sqrt{\frac{(\Gamma_c)^2}{(\omega - \omega_c)^2 + (\Gamma_c)^2}}$$

Putting it all together, we evaluate the voltage-averaged ratio $\frac{E(\omega_t)}{E(\omega_x)} = 0.351$ and $\hbar g_t = 1/4 \cdot 0.351 \cdot 21.01 = 1.84$ meV. Clearly, the optical coupling between guided photons and trions is an order of magnitude weaker than with excitons, due to the smaller oscillator strength and decreased spectral overlap with the guided mode resonance.

Finally, to quantify the impact on the metasurface reflectivity and g_x, we now fit the reflectance spectra with the three-oscillator CMT model by setting $\hbar g_t = 1.84$ meV and leaving g_x as free fitting parameter (Supplementary Fig. S10). From this, we extract $\hbar g_x = 20.93$ meV, corresponding to a relative change in exciton-photon coupling strength of 0.2% compared to the two-oscillator model. At the same time, the mean relative difference in the reflectance across the spectral range of interest and all voltages is only 0.1%. Hence, in this scenario, the use of a two-oscillator CMT with just the excitonic and photonic modes is clearly justified. We emphasize that this is largely due to the fact that excitons and trions are predominantly excited by guided photons instead of being directly excited from free-space. As such, it is not necessary to include the trion as a third oscillator in the CMT analysis, except if the trion oscillator strength would be much stronger and/or the spectral overlap with the guided mode resonance much larger.

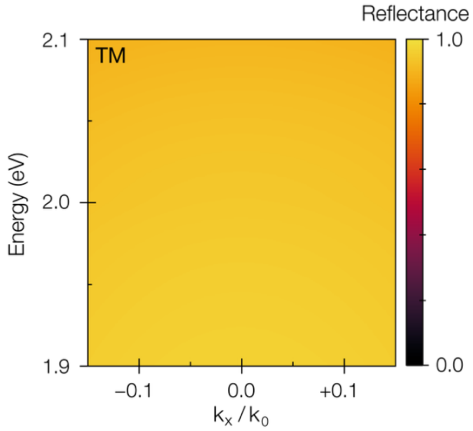

Supplementary Figure S1: Angular dispersion under TM-polarized illumination. Numerically simulated k_x dispersion of the designed modulator under TM-polarized illumination showing mirror-like reflectance in the relevant spectral and angular range.

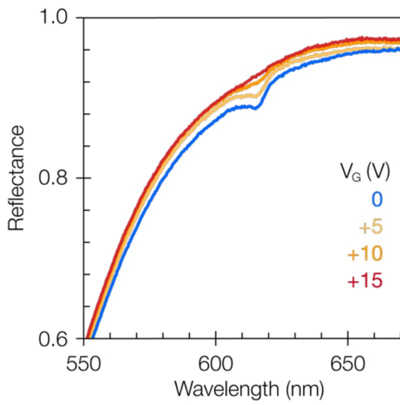

Supplementary Figure S2: Gate-dependent reflectance of a planar device. Experimentally measured reflectance spectra for a bare heterostructure cavity (*i.e.*, without subwavelength grating) as a function of gate voltage (color).

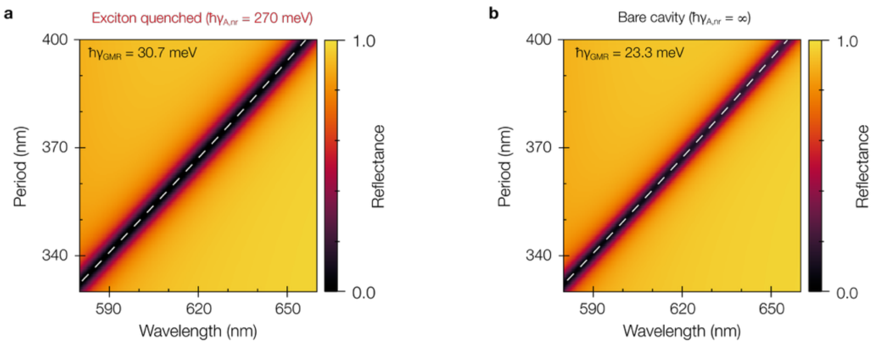

Supplementary Figure S3: Comparison of the modulator in the exciton-quenched state and the bare cavity. (a) Numerically simulated period dependence of the normal-incidence TE-polarized reflectance of the designed modulator in the exciton-quenched state, and (**b)** of the corresponding bare cavity. The fitted dispersion is overlaid on the colormaps (dashed) and the extracted damping rate of the guided-mode resonance (GMR) is indicated.

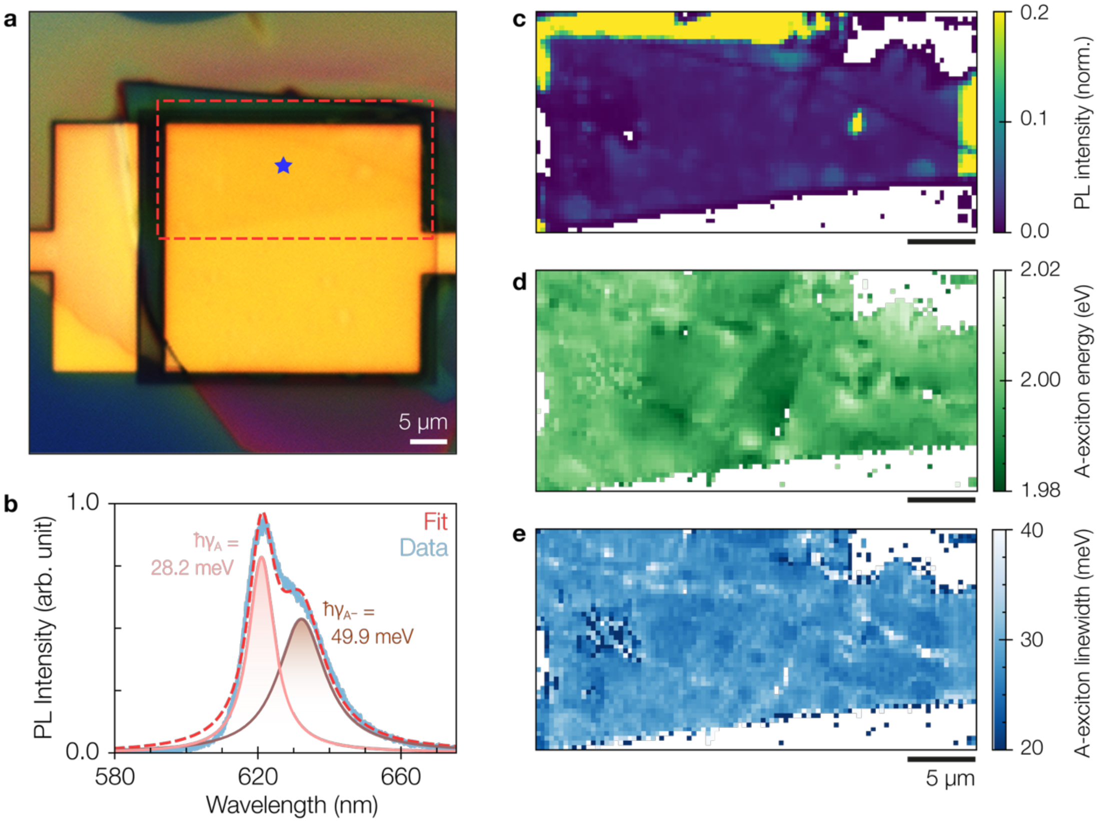

Supplementary Figure S4: Photoluminescence characterization. (a) Brightfield microscope image the fabricated modulator. (**b)** Characteristic PL spectrum (light blue) obtained by averaging a 1 μm^2 spot indicated by the blue star in (a). The data is fitted with a double Lorentzian function (red, dashed) and separated into A-exciton (pink) and negatively charged trion (A^-**,** brown**)** contributions. **(c)** Spatially-resolved photoluminescence (PL) maps of the region (red, dashed) outlined in (a), showing the integrated intensity, **(d)** the fitted A-exciton energy, and **(e)** the fitted linewidth (total decay rate). Scalebars: 5 μm.

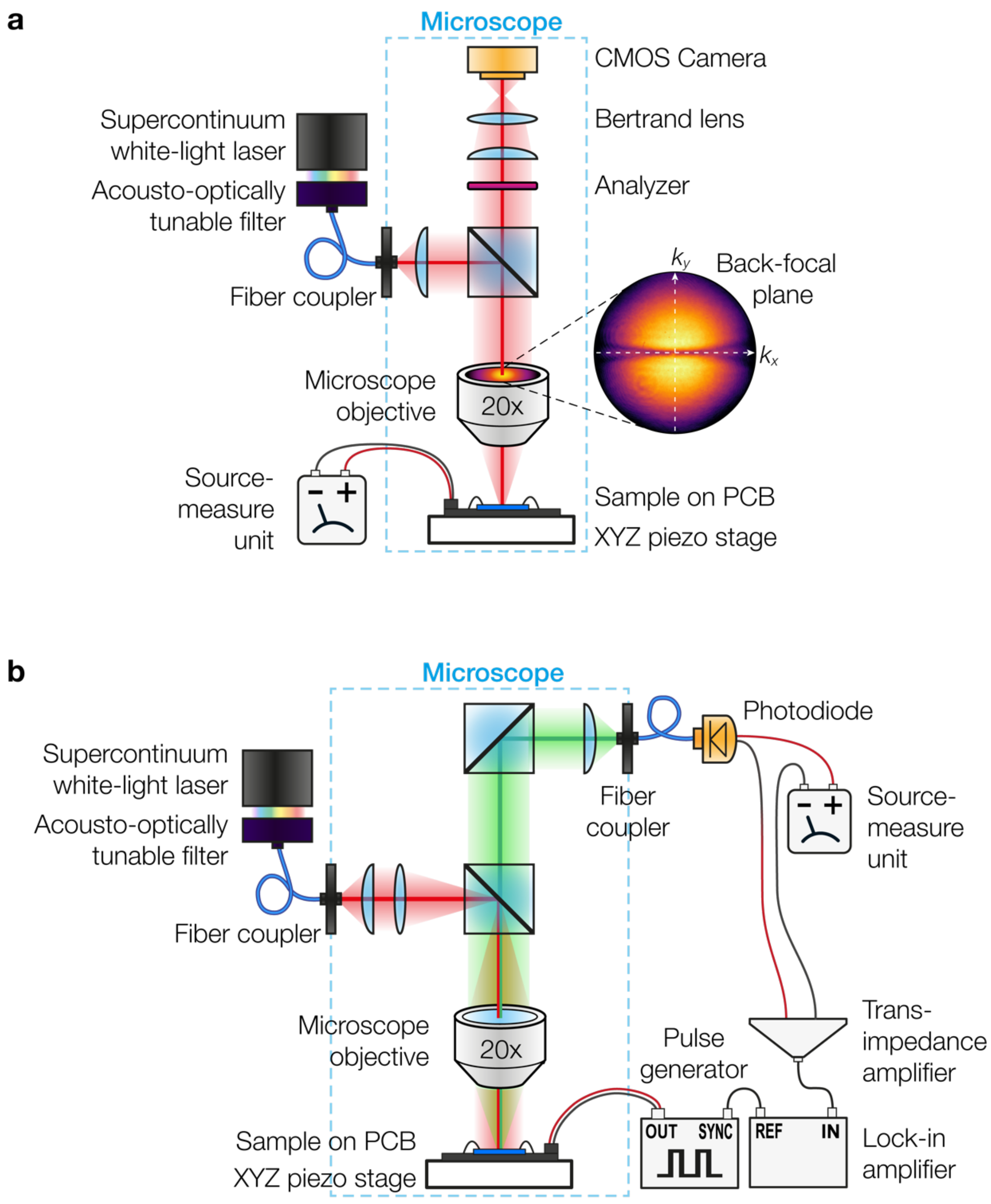

Supplementary Figure S5: Measurement setups for DC and AC modulation experiments. (a) Back-focal plane imaging setup used for angle-resolved reflectance spectroscopy and DC modulation experiments. **(b)** Normal-incidence, spectrally resolved AC reflectance modulation setup.

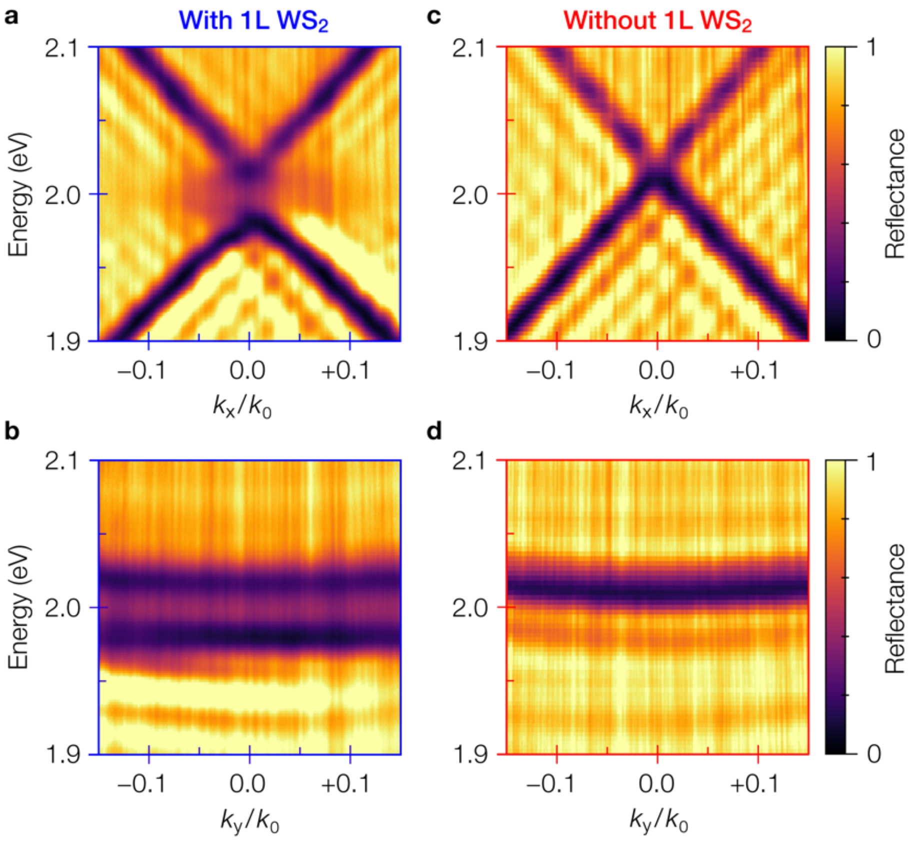

Supplementary Figure S6: Experimental dispersion of the cavity with and without monolayer WS_2. (a) k_x and **(b)** k_y dispersion of the fabricated modulator under TE-polarized illumination. **(c)** k_x and **(d)** k_y dispersion of the empty cavity, measured on a patch of the device that lacks the WS_2 monolayer.

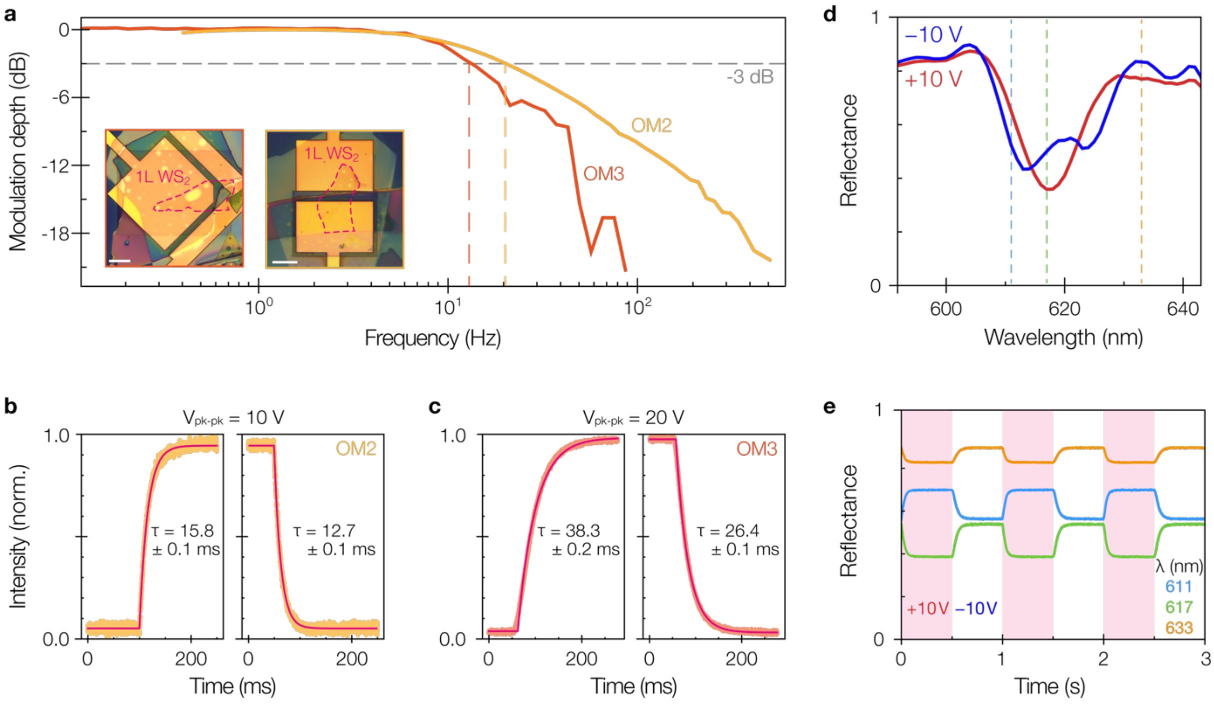

Supplementary Figure S7: AC modulation characteristics of two hybrid-2D modulators. (a) Modulation bandwidth of devices OM2 (yellow) at λ_0 = 631 nm with 3 dB cut-off at f_{-3dB} = 12.8 Hz and OM3 (orange) at λ_0 = 633 nm with f_{-3dB} = 20.2 Hz. Inset shows optical micrographs of OM2 and OM3 with monolayer (1L) WS_2 outlined in magenta. Scalebars: 10 µm. **(b, c)** Corresponding time traces of the rise (left) and fall (right) of the reflected intensity in response to a 1 Hz square-wave modulation signal for devices OM2 (b) and OM3 (c). The rise and fall times obtained by fitting (magenta) and the peak-to-peak driving voltage $V_{\text{pk-pk}}$ are also shown. **(e)** DC reflectance spectra of device OM3 at +10 V (red) and −10 V (blue) and **(e)** reflectance as function of time in response to a 1 Hz pulse at λ_0 = 611 nm (light blue), 617 nm (green) and 633 nm (orange). Colors corresponding to dotted lines in (d).

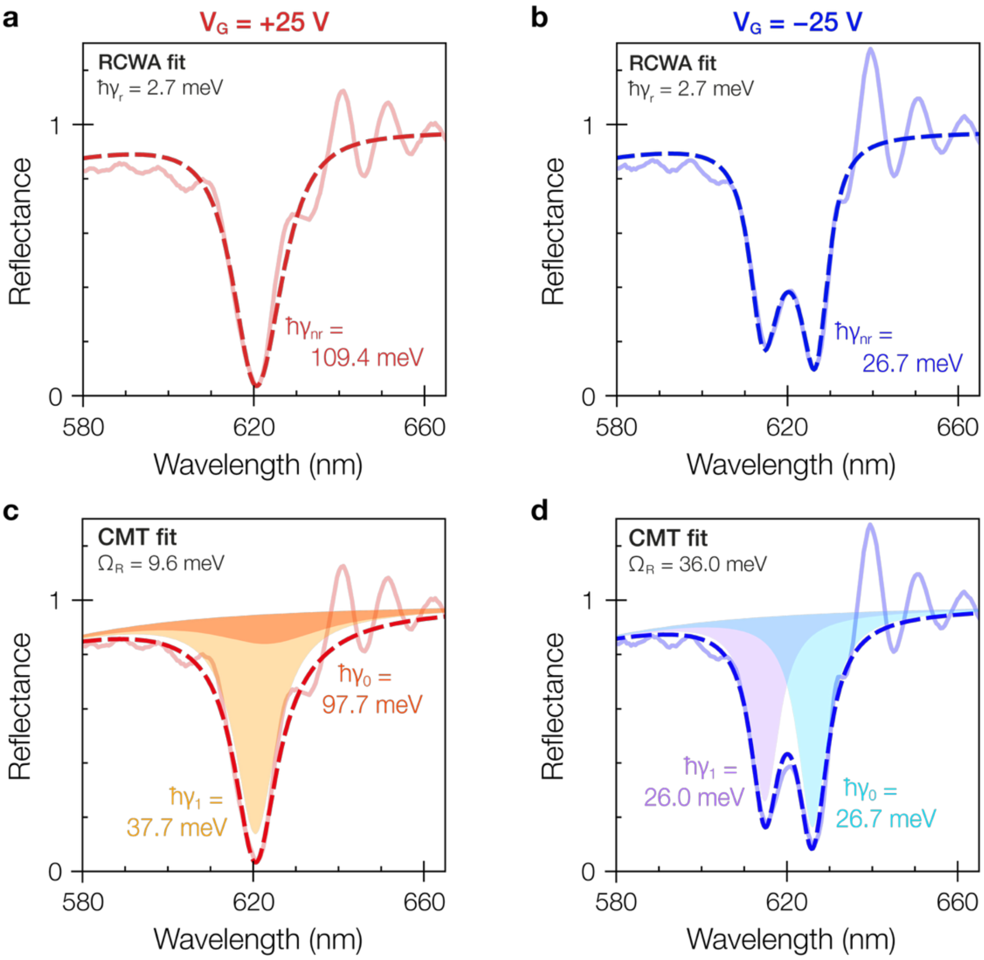

Supplementary Figure S8: Parameter extraction via reflectance fitting. **(a)** Fit (dashed) of the normal-incidence reflectance performed with rigorous coupled-wave analysis (RCWA) for an n-doped (+25 V), and (**b**) a neutral (−25V) monolayer, used to extract the A-exciton energy plus the radiative and nonradiative decay rates, γ_r and γ_{nr}, respectively. **(c)** Fit (dashed) performed with coupled-mode theory (CMT) for an n-doped, and (**d**) intrinsic monolayer, to extract the exciton-photon coupling strength as well as the energies and linewidths (γ_0 and γ_1) of the coupled modes. The CMT fits are offset by a non-resonant background reflectance obtained through RCWA simulations for TM-polarized illumination.

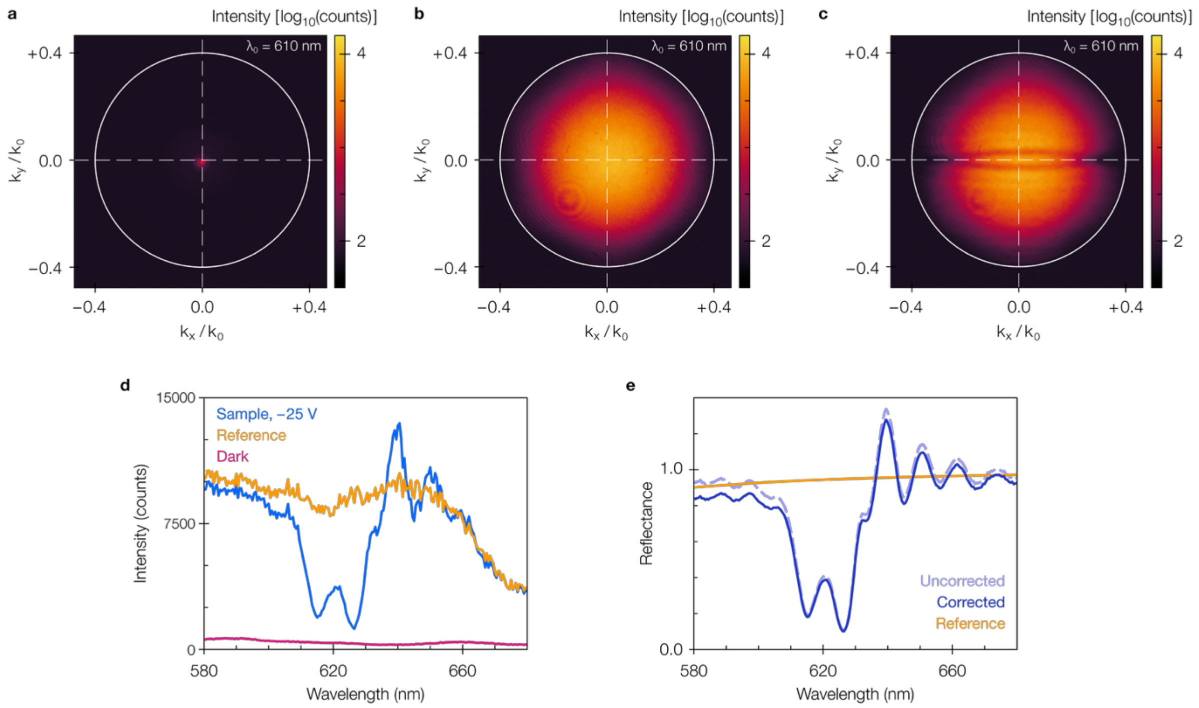

Supplementary Figure S9: Reflectance extraction from back-focal plane images. (a) Back-focal plane image at $\lambda_0 = 610$ nm in absence of a sample (I_{dark}), **(b)** of the gold reference ($I_{reference}$), and **(c)** of the sample at –25 V (I_{sample}). **(d)** The extracted spectral intensities I_{sample} (blue), $I_{reference}$ (yellow), and I_{dark} (magenta) at normal-incidence ($k_x = k_y = 0$). **(e)** Corresponding absolute reflectance of the sample before (dashed, light blue) and after (solid, dark blue) corrected using the RCWA-calculated reference spectrum $R_{reference}$ (yellow).

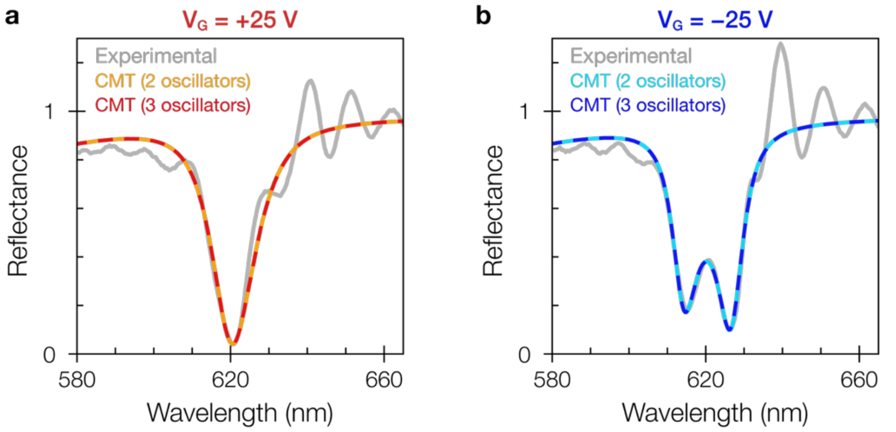

Supplementary Figure S10: Comparison of two- and three-oscillator coupled mode theory models. (a) Fits of the experimental normal-incidence reflectance (grey solid line) for an n-doped (+25 V) monolayer using two-oscillator coupled mode theory (CMT, orange solid line) and three-oscillator CMT (red dashed line). (**b)** Similarly, two-oscillator CMT (light blue solid line) and three-oscillator CMT (dark blue dashed line) fits for a neutral (−25V) monolayer. These fits are used to confirm that the two-oscillator CMT model with only the guided-mode and excitonic resonances is justified, and the contribution from a third trionic oscillator to the reflectance is negligible (mean relative difference of 0.1%).

References

1. Fan, S. H., Suh, W. & Joannopoulos, J. D. Temporal coupled-mode theory for the Fano resonance in optical resonators. *Journal of the Optical Society of America A* **20**, 569 (2003).
2. Haus, H. A. *Waves and Fields in Optoelectronics* (Prentice-Hall, Englewood Cliffs, NJ, 1984).
3. Lien, D. H. et al. Electrical suppression of all nonradiative recombination pathways in monolayer semiconductors. *Science* **364**, 468-471 (2019).
4. Novotny, L. & Hecht, B. *Principles of Nano-Optics* (Cambridge University Press, Cambridge, 2012).
